# Short-term exposure to ammonium sulfate modifies ice nucleation by alpha-alumina but not organic monolayers or microplastics

**DOI:** 10.1039/d5ea00157a

**Published:** 2026-07-08

**Authors:** Lian Pharoah, Teresa M. Seifried, Gren N. Patey, Anita Lam, Allan K. Bertram

**Affiliations:** a Department of Chemistry, University of British Columbia Vancouver British Columbia Canada V6T 1Z1 bertram@chem.ubc.ca

## Abstract

Accurate predictions of ice cloud formation require understanding how ammonium sulfate affects the freezing behavior of ice-nucleating substances (INSs). This knowledge is also essential for evaluating the reliability of a freezing assay used to identify ice-nucleating mineral dusts in atmospheric samples. Here, we used a droplet freezing technique to investigate the effects of short-term exposure (minutes) to ammonium sulfate on six INSs: two α-alumina samples (submicrometer, < 1 µm, and supermicrometer, > 1 µm), two alcohol monolayers (C_22_H_45_OH and C_30_H_61_OH), and two microplastics (polyethylene terephthalate and low-density polyethylene). Exposure to ammonium sulfate did not change the ice-nucleating properties of the alcohol monolayers or microplastics. In contrast, submicrometer α-alumina exhibited enhanced ice-nucleating ability following ammonium sulfate exposure, while the supermicrometer α-alumina showed no change, despite similar bulk composition. This size-dependent response is consistent with differences in surface hydroxy protonation states. More deprotonated, negatively charged surface hydroxy groups can attract ammonium cations, modifying interfacial hydrogen-bonding networks and stabilizing nascent ice clusters. These findings should be considered when evaluating submicrometer α-alumina for stratospheric aerosol injection as a solar management strategy. In addition, our results support the use of ammonium sulfate-based freezing assays for identifying ice-nucleating mineral components in atmospheric samples.

## Abstract 2

Short-term exposure to ammonium sulfate enhances ice nucleation by submicrometer α-alumina but not by alcohol monolayers or microplastics, supporting ammonium sulfate assays for identifying mineral ice nucleators.

## Graphical abstract

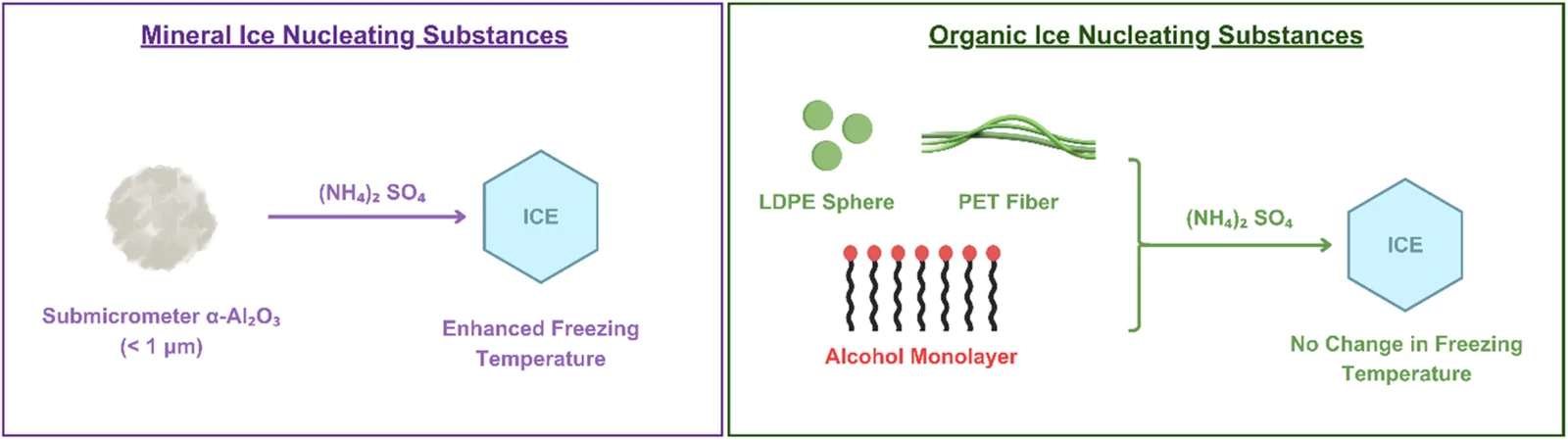

**Environmental significance**

Ice-nucleating substances (INSs) influence our climate by participating in various atmospheric processes, the extent of which is related to their freezing behavior. During atmospheric transport, INSs can become coated or mixed with solutes like ammonium sulfate, which modifies the freezing behavior of INSs. Our α-alumina results support previous assumptions of enhanced freezing behavior observed for mineral dust INSs exposed to ammonium sulfate, but highlight the impact of surface structures on the freezing behavior of INSs. Our findings also support the use of ammonium sulfate in freezing assays for compositional analysis. Overall, our results provide additional context on the effect of ammonium sulfate on the freezing behavior of INSs, which allows for accurate predictions of atmospheric processes that influence the climate.

## 1 Introduction

Atmospheric ice formation occurs either through homogeneous or heterogeneous ice nucleation. Homogeneous nucleation involves the spontaneous freezing of pure water droplets and requires temperatures near −38 °C.^1,2^ In contrast, heterogeneous nucleation is triggered by the presence of ice nucleating substances (INSs), enabling ice formation at warmer temperatures and requiring smaller critical ice nuclei compared to homogeneous nucleation.^1–7^ Most ice formation in clouds is initiated by heterogeneous nucleation on INSs.^7–10^ Consequently, INSs influence the Earth's climate by impacting the phase state of clouds, which can alter cloud lifetimes, cloud optical properties, and precipitation patterns.^11–14^

Two major types of atmospheric INSs are organic particles and mineral dusts. Organic INSs include bacteria,^15–17^ fungal spores,^18–22^ pollen,^23,24^ microplastics,^25–33^ and cell exudates.^34–38^ Mineral dust INSs include *e.g.* mica, feldspar, kaolinite, and quartz.^39–44^ Another type of atmospheric INS is metals from industrial sources, which could explain the observed increased freezing temperatures of clouds impacted by anthropogenic pollution.^45,46^

During transport, INSs can become coated with or mixed with solutes such as sodium chloride and ammonium sulfate.^47–49^ Solutes can affect ice nucleation in two ways: (1) by lowering water activity, which results in freezing point depression,^50^ and (2) by modifying the surface properties of the INS, which could either enhance or inhibit ice nucleation.^42–44,51–56^ Among solutes, ammonium sulfate is particularly important because it is abundant in the atmosphere, produced through SO_2_ oxidation and neutralization by ammonia. Given its prevalence, ammonium sulfate is often mixed with INSs. To accurately predict atmospheric ice formation, it is important to quantify how ammonium sulfate exposure affects the freezing behavior of INSs.

The effect of ammonium sulfate on the freezing properties of several types of minerals have been investigated.^42–44,52,57^ This work has shown that ammonium cations can enhance the ice-nucleating ability of certain minerals.^42,55,58^ Minerals that exhibit enhanced ice nucleation ability in the presence of ammonium cations include K-rich feldspar, kaolinite and montmorillonite.^42,55,58^ However, some atmospherically relevant minerals have not yet been examined for their response to ammonium sulfate. The mechanism underlying this enhancement has been explored in several studies. For K-feldspar, ammonium cations enhance ice nucleation through ion exchange and surface adsorption.^42,57,59,60^ Chen *et al.* confirmed the ion-exchange mechanism using ammonium and aminium cations.^58^ In contrast, minerals like kaolinite, which do not undergo ion exchange, still show enhancement through ammonium adsorption that strengthens surface hydrogen bonding.^42–44^ Whale *et al.* proposed that the ice nucleation ability of many inorganic particles originate from polar or charged surface sites that orient adjacent water molecules.^59^ They further suggest that ammonium cations enhance ice nucleation activity by disrupting thermodynamically unfavorable hydrogen-bond arrangements within nascent ice clusters that form at these water-structuring interfaces.^61^

The effect of ammonium sulfate on the ice nucleation ability of organic INSs has been examined in multiple studies.^52,57,62,63^ For example, Worthy *et al.* investigated the effects of short-term exposure to ammonium sulfate for a wide range of organic INSs including, humic acid, bacteria, fungi and sea surface microlayer substance, and found no enhancement in ice nucleation.^55^ Similarly, Cantrell *et al.* studied several alcohol monolayers (C_16_H_33_OH, C_17_H_35_OH, C_25_H_51_OH, and C_30_H_61_OH) and also observed no change in freezing temperature.^64^ Busse *et al.* examined the effects of long-term exposure (days) to ammonium sulfate on various microplastics – polypropylene (PP), polyethylene terephthalate (PET), polyvinyl chloride (PVC), and low-density polyethylene (LDPE) – and reported a decreasing ice-nucleating ability for PP and PET, but an increase for PVC and LDPE.^27^ Although several studies have examined this topic, the overall effect of ammonium sulfate on the freezing behavior of atmospherically relevant organics remains incompletely understood.

A freezing assay was recently proposed and used by our group to identify specific types of mineral dust in atmospheric samples based on their response to ammonium sulfate exposure.^42–44,52,57^ In this method, an increase in freezing temperature after adding ammonium sulfate indicates the presence of ice-nucleating minerals. The ammonium sulfate assay is straightforward and can be implemented using instrumentation available in many laboratories, which makes the method advantageous. The method assumes that short-term exposure (on the order of a few minutes) to ammonium sulfate enhances freezing for ice-nucleating minerals (*e.g.*, K-feldspar, kaolinite and montmorillonite) but does not enhance freezing of organic INSs. To determine which mineral INSs this assay applies to and to further assess its accuracy and reliability, additional studies are needed on both mineral and organic INSs. Such investigations are necessary to validate the assay as a diagnostic tool for identifying ice-nucleating minerals within atmospheric samples.

In this work, we used a droplet freezing technique to investigate the effect of short-term exposure to ammonium sulfate on six different INSs: two α-alumina particle types (submicrometer, < 1 µm, and supermicrometer, > 1 µm), two alcohol monolayers (C_22_H_45_OH and C_30_H_61_OH), and two microplastics (PET fibers and LDPE spheres). This study is the first to investigate the effect of ammonium sulfate on aluminum oxide particles and the first to examine how short-term exposure (a few minutes) to ammonium sulfate influences the ice-nucleating properties of two types of microplastics. In addition, our measurements are the first to assess the effect of ammonium sulfate on the freezing behavior of the C_22_H_45_OH alcohol monolayer. These results improve understanding of how ammonium sulfate interacts with atmospherically relevant ice-nucleating substances and help evaluate the reliability of freezing assays used to identify mineral dust in atmospheric samples. The justification for each of the six ice-nucleating materials is expanded upon below.

α-Alumina is commonly used as a proxy for corundum, a naturally occurring rock-forming mineral. α-Alumina has also been studied as model systems for understanding the ice-nucleating properties of atmospheric mineral dust particles.^65–67^ In addition, alumina particles are being considered for stratospheric aerosol injection to cool the Earth's climate.^68,69^ The required injection amount is on the order of a few teragrams per year, and the atmospheric lifetime of alumina particles in the stratosphere is expected to be approximately one year.^69^ In the stratosphere, alumina particles would become coated with sulfuric acid.^70^ After transport to the troposphere, the sulfuric acid can be neutralized by ammonium gas to form ammonium sulfate,^56,71^ after which the coated alumina particles could nucleate ice in clouds.

Alcohol monolayers have previously been used as a model system for studying organic INSs with exposed hydroxylated surfaces.^72,73^ Their components have been detected in marine aerosols and in the ocean surface microlayer, and are likely introduced into the atmosphere through bubble bursting.^34,74,75^

Measured atmospheric microplastic concentrations span several orders of magnitude, ranging from <1 to >10^2^ particles per liter of air.^76,77^ PET and LDPE microplastics represent two major and atmospherically relevant classes of microplastics.^77,78^ PET fibers are consistently reported as a dominant form of airborne microplastics by number, largely originating from synthetic textiles.^79,80^ Polyethylene is among the most commonly detected polymers in atmospheric microplastics and is widely used in packaging and consumer products.^81^

## 2 Methodology

### 2.1 Samples

Two α-alumina powders, referred to here as submicrometer and supermicrometer samples, were purchased from Alfa Aesar. Based on information from the manufacturer, the particle diameters of the submicrometer and supermicrometer samples were < 1.0 µm and 20–50 µm, respectively. Based on previous Brunauer–Emmett–Teller (BET) surface area analysis of the same α-alumina powders, the surface areas of the supermicrometer and submicrometer samples are 5 m^2^ g^−1^ and 3 m^2^ g^−1^, respectively.^82^

Scanning electron microscopy (SEM) measurements were carried out to confirm the size and determine the morphology of the α-alumina powders. Table S1 includes details on the instrument used for SEM measurements, experimental conditions and sample preparation. The resultant SEM images are shown in Fig. S1 for the supermicrometer (Fig. S1a–c) and submicrometer (Fig. S1 d–f) α-alumina powders. The SEM images confirm that the supermicrometer particles are several micrometers in size. The SEM images also show that the surface of the supermicrometer particles are highly porous and composed of what appears to be aggregates of smaller structures. These submicrometer features give the larger particles a rough, high-surface-area morphology.

X-Ray diffraction (XRD) measurements were carried out to determine the composition of the α-alumina powders. Table S2 includes details on the instrument used for the XRD measurements, experimental conditions, database collection, and sample preparation. The resultant XRD patterns of the submicrometer and supermicrometer α-alumina powders are shown in Fig. S2 and S3, respectively. XRD Rietveld analyses for the submicrometer α-alumina powders are shown in Fig. S4 and S5. XRD Rietveld analysis for the supermicrometer α-alumina powder is shown in Fig. S6. XRD analysis revealed that the submicrometer sample contained 95% α-Al_2_O_3_ and 5% Al_18_B_4_O_33_ and the supermicrometer sample contained 100% α-Al_2_O_3_.

The C_22_H_45_OH and C_30_H_61_OH alcohols were purchased from Sigma-Aldrich and had purities of 98.0% and ≥ 98.0%, respectively. Chloroform, which was used as a spreading solvent for the alcohols, was purchased from Fisher Scientific, HPLC grade, and had a purity of ≥ 99.5%. Ammonium sulfate was purchased from Fisher Scientific and had a purity of ≥ 99.0%.

The PET fibers were obtained from Cotton Incorporated (North Carolina), which provides manufactured standard fiber textiles for environmental assessments and toxicity studies. The PET fibers were cut into small pieces (∼1.0 cm) and then ground using a Wiley Mill, as done in a previous ice nucleation study.^26^ The LDPE spheres were purchased from Nanochemazone (Leduc, AB, Canada). The size of PET fibers was approximately 20 µm × 300 µm, and the LDPE spheres had an approximate diameter of 55 µm.^26^

### 2.2 Droplet freezing experiments

**Fig. 1:**
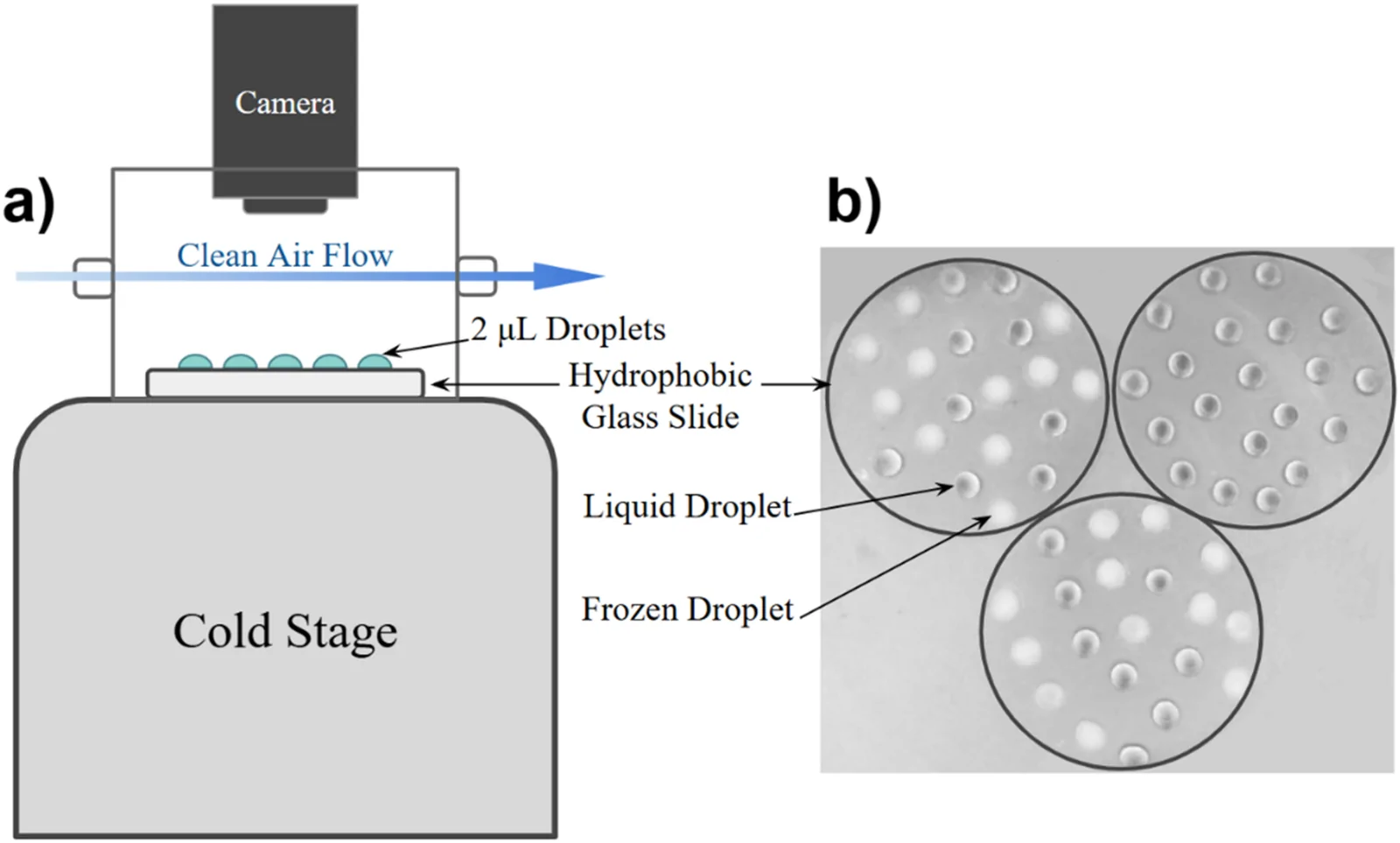
(a) Experimental setup for freezing experiments, and (b) snapshot of the video taken by the camera during the freezing of droplets coated with C_30_H_61_OH taken approximately at −11.2 °C.

We examined the effect of short-term exposure (a few minutes) to ammonium sulfate on the ice nucleation ability of α-alumina powders, fatty alcohol monolayers and microplastics with a droplet freezing technique.^83^ This method has been previously applied to study ice nucleation by organic substances and mineral dusts.^26,27,51^ In general, this technique utilizes a cold stage on which the freezing of 2 µL droplets is studied. The cold stage is covered with a transparent chamber through which a small constant flow of clean air is applied during a freezing experiment to prevent condensation (Fig. 1a). The chamber is equipped with a digital camera located above the cold stage to record a video of the droplet freezing events.

For the α-alumina freezing experiments, we first prepared suspensions of α-alumina in Milli-Q water (specific resistivity 18.2 MΩ cm^−1^, 25 °C). A concentration of 0.1 wt% was used for the submicrometer α-alumina, and 0.3 wt% for the supermicrometer α-alumina. These concentrations were selected to promote freezing temperatures warmer than that of pure water. After stirring the suspensions for 12 hours, 1 µL droplets of the suspensions were pipetted onto hydrophobic siliconized glass slides (18 mm diameter, HR30239; Hampton Research, Aliso Viejo, CA) and placed on the cold stage. An additional 1 µL droplet of either Milli-Q water or aqueous ammonium sulfate solution was added resulting in a 2 µL droplet with or without (NH_4_)_2_SO_4_. The sample preparation for microplastics were conducted in analogy with the sole difference of creating 0.02 wt% suspension of both PET fibers or LDPE spheres.

In the experiments above, the INSs were prepared in pure water suspension and only exposed to ammonium sulfate after a 1 µL droplet of aqueous ammonium sulfate was added to the 1 µL droplet of the INSs suspended in pure water. The time that the INSs were exposed to aqueous ammonium sulfate prior to the freezing experiments was only a few minutes. The concentrations of ammonium sulfate to which the INSs were exposed ranged from 2 to 200 mM. The corresponding water activities for these concentrations were 0.999 and 0.992.

To prepare droplets with alcohol monolayers at the air–water interface, we followed a procedure similar to that described in previous studies.^64,84–87^ First, 2 µL droplets of either Milli-Q water or ammonium sulfate solution were pipetted onto hydrophobic siliconized glass slides (22 mm diameter). The concentration of ammonium sulfate solution ranged from 2 to 200 mM, and the water activity of the ammonium sulfate solution ranged from 0.999 to 0.992. The alcohol monolayer was applied using chloroform as the spreading solvent. At least two minutes were allowed for the spreading solvent to evaporate before the freezing measurements. The amount of alcohol added corresponds to ∼20 monolayers at equilibrium spreading pressure, as calculated from the alcohol concentrations, the exposed droplet surface area, and previously reported packing densities of the alcohols at equilibrium spreading pressure.^84–87^

After the droplets with the INSs were prepared, freezing experiments were immediately carried out. During the freezing experiments, the cold stage was cooled at 3 °C min^−1^ until all droplets were observed to be frozen. For each sample type investigated, background freezing experiments were also carried out using pure water droplets to determine the background freezing level of water in the experiments. Between three and five replicate background measurements were performed for each sample type. The recorded video and temperature data were imported into a MATLAB script, which assigned a freezing temperature for each droplet.^88^

All freezing data is corrected for freezing point depression using the following equation:^50^

1 Δ*T*_f_ = *i* × *K*_f_ × *m*_solute_,

where Δ*T*_f_ is the freezing point depression, *i* is the van't Hoff factor (we assume *i* = 3 since only low concentrations of ammonium sulfate is used), *K*_f_ is the cryoscopic constant for water (1.86 °C kg mol^−1^), and *m*_solute_ is the molality of ammonium sulfate in the 2 µL droplets.

From the freezing experiments, we calculated the fraction of frozen droplets as a function of temperature (*f*_ice_(*T*)). In addition, to quantify the effect of ammonium sulfate on the freezing properties of α-alumina, alcohol monolayers and microplastics, we calculated the change in median freezing temperature of the droplets due to the addition of ammonium sulfate, Δ*T*_50_ using the following equation:

2 Δ*T*_50_ = *T*_50,(NH_4_)_2_SO_4__ − *T*_50,H_2_O_

where *T*_50,(NH_4_)_2_SO_4__ is the average median freezing temperature of droplets containing the α-alumina powders, alcohol monolayer, or microplastics with the addition of ammonium sulfate (after correcting for freezing point depression), and *T*_50,H_2_O_ is the average median freezing temperature of droplets containing the α-alumina, alcohol monolayer, or organic microplastics in pure water. Each *T*_50_ value represents an average over all the days the measurements were recorded.

## 3 Results & discussion

### 3.1 α-Alumina particles

We conducted freezing measurements of water droplets and aqueous ammonium sulfate droplets containing supermicrometer and submicrometer α-alumina particles. For pure water droplets containing supermicrometer and submicrometer α-alumina particles, *T*_50_ values were −20.8 °C ± 0.3, (*p* = 0.01) and −21.1 °C ± 0.8 (*p* = 0.01), respectively (Fig. 3). These values are consistent with the median freezing temperature reported by Chong *et al.* for water droplets containing α-alumina (*T*_50_ = −20.8 °C).^89–91^

**Fig. 2:**
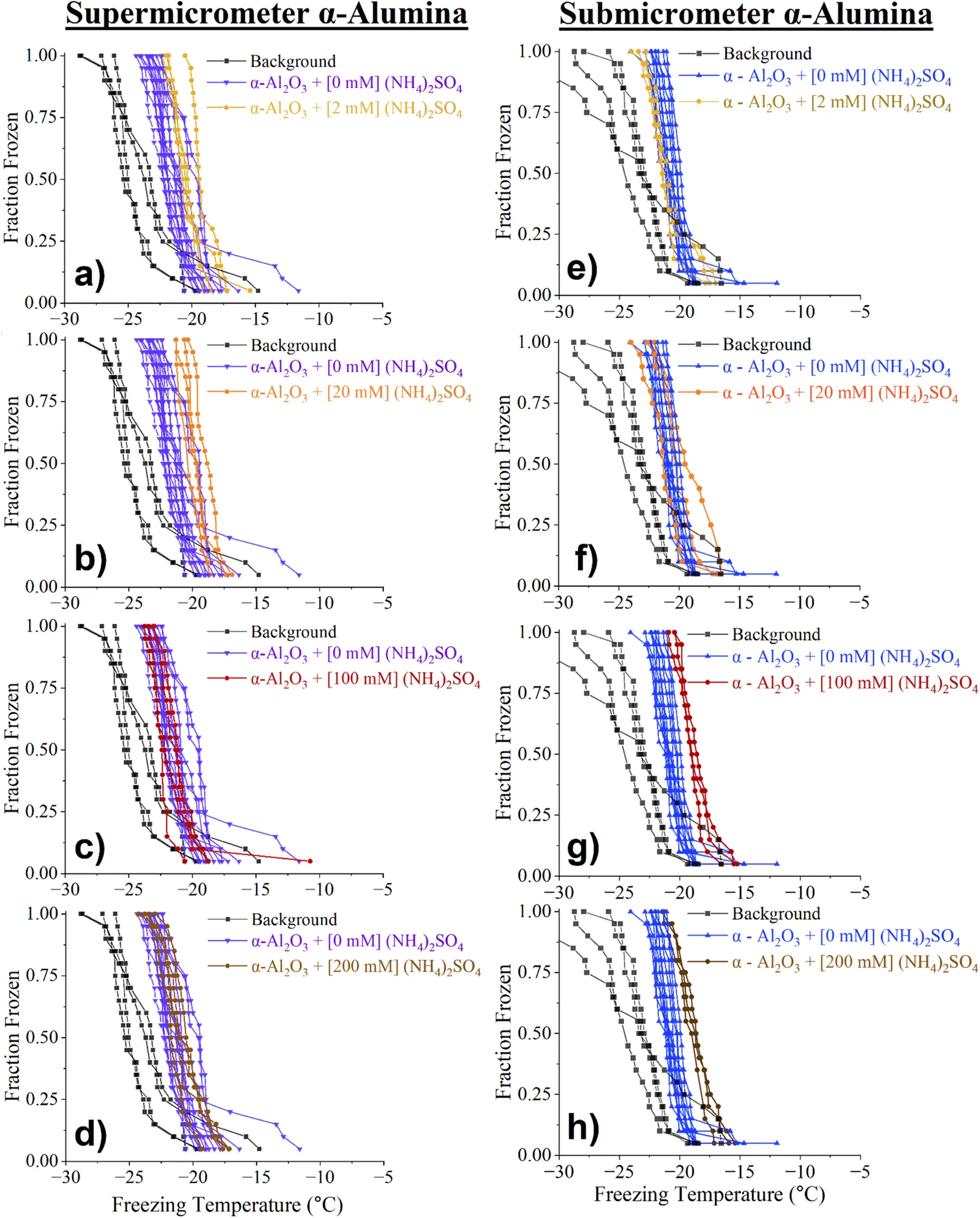
Freezing curves for aqueous ammonium sulfate droplets containing supermicrometer (left panel) and submicrometer α-alumina (right panel): (a and e) 2 mM ammonium sulfate, (b and f) 20 mM ammonium sulfate, (c and g) 100 mM ammonium sulfate, and (d and h) 200 mM ammonium sulfate. For comparison, the curves for Milli-Q water droplets with supermicrometer and submicrometer α-alumina at 0 mM ammonium sulfate are shown in purple and blue, respectively. The freezing curves for the background (water with 100 mM ammonium sulfate) are shown in gray. Freezing temperatures are corrected for freezing point depression.

**Fig. 3:**
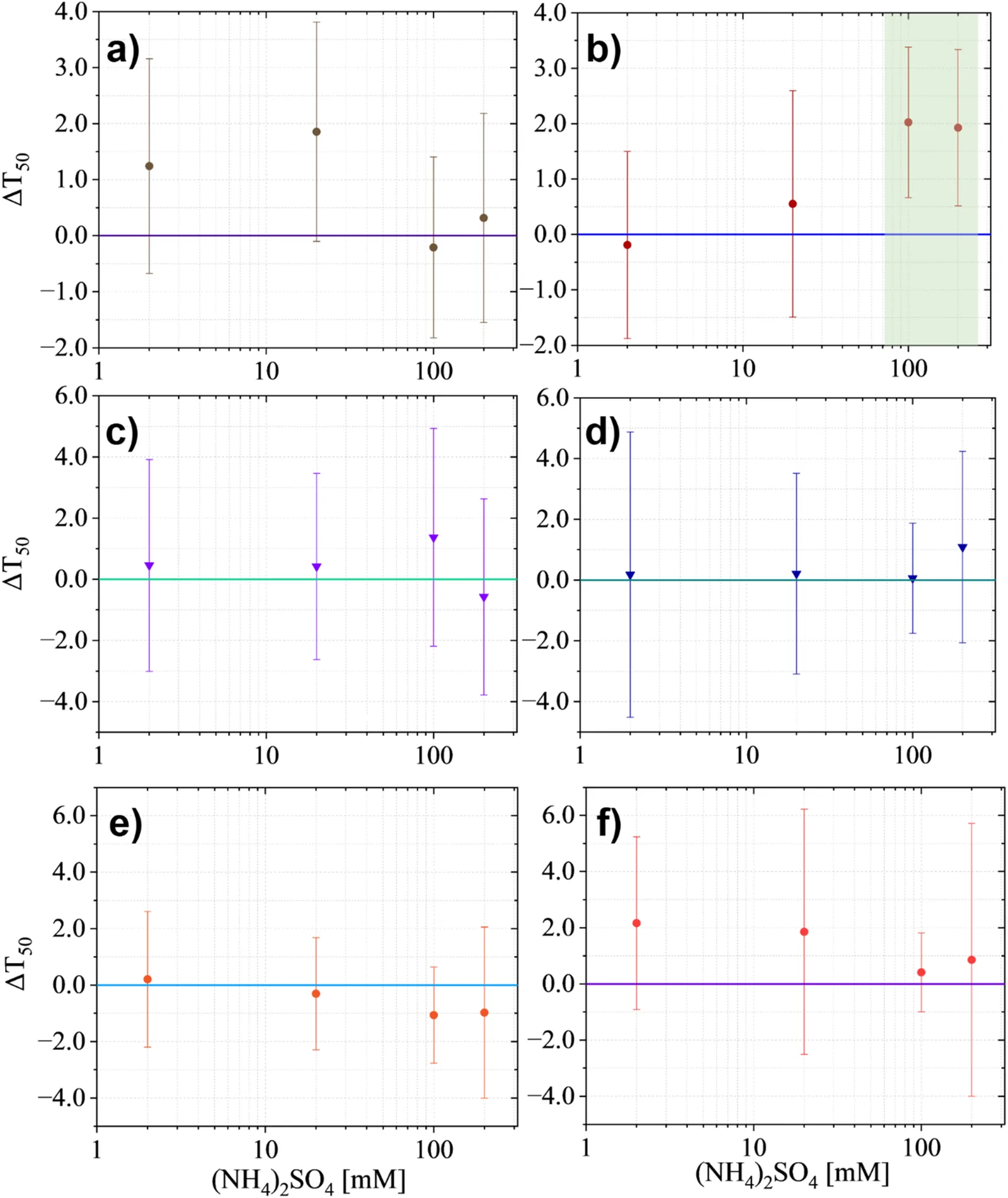
Change in median freezing temperature (Δ*T*_50_) for (a) supermicrometer and (b) submicrometer α-alumina samples, (c) C_22_H_45_OH and (d) C_30_H_61_OH alcohol monolayers, and (e) PET fibers and (f) LDPE spheres, at varying ammonium sulfate concentrations. The error bars represent 99% CI based on a Student's *t* distribution. Freezing temperatures are corrected for freezing point depression. The shaded region in (b) indicates concentrations where Δ*T*_50_ values are statistically different from zero at the 99% confidence level (*p* < 0.01).

For aqueous ammonium sulfate droplets containing supermicrometer α-alumina particles, the freezing curves overlap with those of pure water droplets containing the same particles, after correcting for freezing point depression (Fig. 2a–d). Across all ammonium sulfate concentrations, the Δ*T*_50_ values for supermicrometer α-alumina are indistinguishable from zero within the measurement uncertainties (Fig. 3a), indicating the effect of ammonium sulfate on the freezing properties of the supermicrometer α-alumina particles was smaller than the measurement uncertainties.

For aqueous ammonium sulfate droplets containing submicrometer α-alumina particles, the freezing curves overlapped with those for pure water droplets containing the same particles at low ammonium sulfate concentrations (2 mM and 20 mM), but diverged at higher concentrations (100 mM and 200 mM) (Fig. 2e–h). In addition, the Δ*T*_50_ values for submicrometer α-alumina were indistinguishable from zero within uncertainty at 2 mM and 20 mM. However, at 100 mM and 200 mM, the Δ*T*_50_ values were +1.9 °C ± 0.9 and +1.8 °C ± 0.7, respectively (Fig. 3b), indicating that ammonium sulfate increases the ice nucleation temperature of submicrometer α-alumina at the highest concentrations examined. It is not surprising that Δ*T*_50_ values statistically different from zero are only observed at the highest ammonium sulfate concentrations studied (100 mM and 200 mM) and not at the lowest concentrations (2 mM and 20 mM). Previous studies have shown that the magnitude of the Δ*T*_50_ values increases with ammonium sulfate concentration, with the largest effects expected at the highest concentrations.

**Fig. 4:**
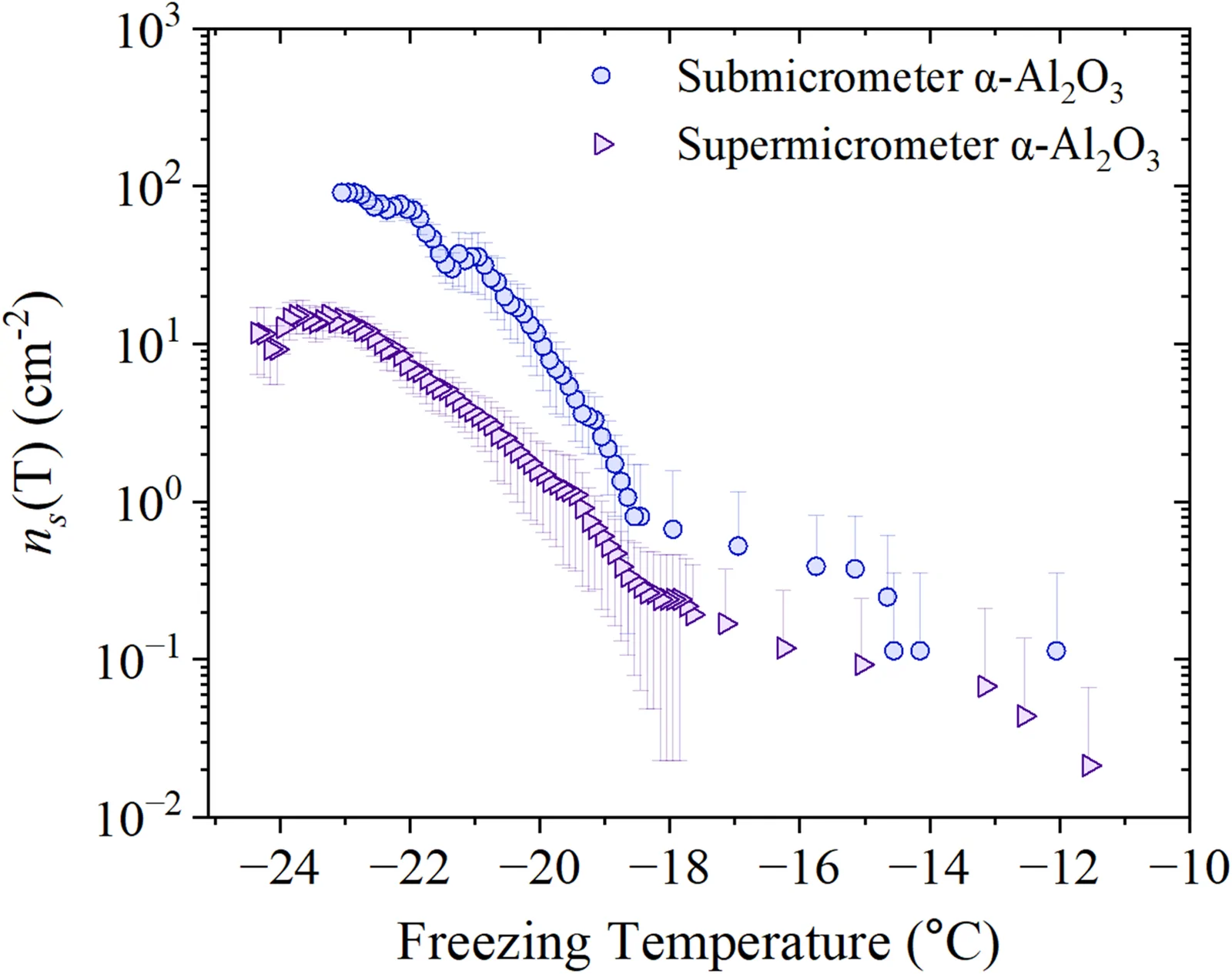
Ice nucleation active site densities, *n*_s_(*T*), for the submicrometer (blue) and supermicrometer (purple) α-alumina samples. Error bars denote the 99% CI (*p* < 0.01) of *n*_s_(*T*) at temperature *T*, calculated using Student's *t*-distribution.

Our experiments show that the freezing properties of supermicrometer and submicrometer α-alumina particles responded differently to ammonium sulfate. To further investigate differences between the supermicrometer and submicrometer particles, we compared the number of ice nucleation sites per surface area for the two size classes. *n*_s_(*T*), was calculated from the freezing data using the following equation:^66^3
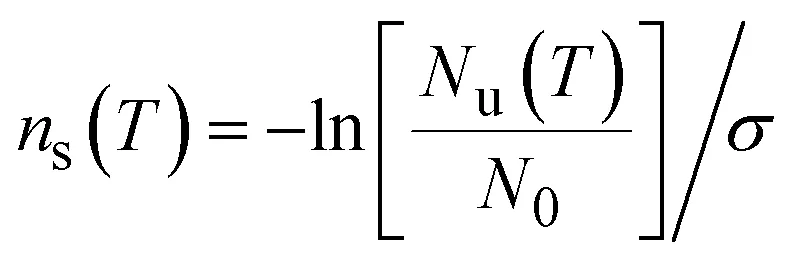
where *N*_u_(*T*) is the number of unfrozen droplets at temperature *T*, *N*_0_ is the number of total droplets, and *σ* is the average surface area of α-alumina particles per droplet. The average surface areas were determined from the mass and the BET surface area of the α-alumina particles in the droplets of the particles (discussed in Section 2.1.). Based on the freezing results and the BET surface areas, the submicrometer α-alumina powder has higher *n*_s_(*T*) values than the supermicrometer α-alumina powder in the temperature range of −20 to −24 °C (Fig. 4). These results illustrate that the surface properties of the two samples differ.

Overall, our results show that supermicrometer and submicrometer α-alumina powders exhibit distinct ice nucleation abilities and respond differently to ammonium sulfate, even though they share a similar bulk composition. These differences are most likely due to variations in surface properties between the two samples. The samples may possess different crystal faces or types of surface imperfections—such as defects, steps, or edges—that differ in both their ice-nucleating activity and their sensitivity to ammonium sulfate. In addition, the surface compositions may differ substantially. The submicrometer sample contained approximately 5% Al_18_B_4_O_33_, and it is possible that this impurity is present at the surface, where it could facilitate ice nucleation. Further studies on the freezing properties of Al_18_B_4_O_33_ are needed to test this possibility. Regardless, our findings emphasize that surface properties play a critical role in determining both ice nucleation behavior and the response to ammonium sulfate.

Several studies have suggested that the enhanced ice nucleation ability of K-feldspar in the presence of ammonium cations is due to ion exchange between ammonium and potassium in the K-feldspar crystal lattice.^42,54,58^ This exchange exposes N–H groups at the K-feldspar surface that can hydrogen bond with ice and promote ice nucleation. This mechanism cannot explain the enhanced ice nucleation ability of submicrometer α-alumina samples after short-term exposure to ammonium, since alumina does not contain cations in its crystal lattice that can undergo ion exchange with ammonium.

Whale *et al.*^59^ proposed a more general mechanism in which ice nucleation on many types of inorganic particles originates from polar or charged surface sites that orient adjacent water molecules. They further suggest that ammonium enhances ice nucleation by disrupting thermodynamically unfavorable hydrogen-bond arrangements within nascent ice clusters that form at these structured interfaces. Taken together, this general mechanism suggests that inorganic particles that nucleate ice at polar or charged surface sites will be more sensitive to ammonium cations. In these cases, ammonium cations may readily partition to the polar or charged surfaces and enhance ice nucleation. In contrast, ammonium cations may partition less readily to non-polar or uncharged surface sites, and consequently the ice nucleation properties of such sites may be less sensitive to the presence of ammonium cations.

In aqueous solution, aluminum oxide surfaces are terminated by hydroxy groups. These surface hydroxy groups can be dual-protonated, monoprotonated, or deprotonated depending on their p*K*_a_ values and solution pH:

4 (Al)_2_OH^+^_2_ ⇋ (Al)_2_OH + H^+^ (*K*_a_1__)

5 (Al)_2_OH ⇋ (Al)_2_O^−^ + H^+^ (*K*_a_2__)

The p*K*_a_ values determine both the protonation state of the surface hydroxy groups and overall surface charge. For example, the α-alumina (0001) surface has reported p*K*_a_ values^92,93^ of p*K*_a_1__ = 3 and p*K*_a_2__ = 9.7 (for eqn (4) and (5), respectively). Thus, the surface is predominately dual-protonated at pH < 3, monoprotonated between 3 < pH < 9.7, and deprotonated at pH > 9.7.

It has been shown that p*K*_a_ values differ between the α-alumina (0001) single crystal surface and α-alumina powders, likely due to differences in the types and distributions of surface hydroxy groups.^94^ It is also possible that submicrometer and supermicrometer α-alumina particles possess different p*K*_a_ values.^92,95^ If the p*K*_a_2__ of the submicrometer particles is lower than that of the supermicrometer particles, then at a given pH the submicrometer particles could be more deprotonated and therefore more negatively charged.

Following the mechanism of Whale *et al.*,^59^ a more negatively charged submicrometer surface would attract more ammonium cations. These ammonium ions could disrupt unfavorable hydrogen-bond arrangements in emerging ice clusters, thereby enhancing ice nucleation. We therefore suggest that the different responses of ice nucleation temperatures to ammonium sulfate observed for submicrometer and supermicrometer α-alumina particles may arise from differences in the p*K*_a_ values of their surface hydroxy groups.

### 3.2 Alcohol monolayers (C_22_H_45_OH and C_30_H_61_OH)

The median freezing temperatures for pure water droplets coated with C_22_H_45_OH and C_30_H_61_OH monolayers, were −13.0 °C ± 0.3 (*p* = 0.01) and −10.6 °C ± 0.6 (*p* = 0.01), respectively (Fig. 3c and d). The warmer freezing temperatures of pure water droplets coated with C_30_H_61_OH compared to C_22_H_45_OH can be explained by the increased number of methylene–methylene interactions for C_30_H_61_OH compared to C_22_H_45_OH resulting in more crystalline monolayers and a better lattice match with ice.^84–86^

Our measured median freezing temperatures for pure water droplets coated with C_22_H_45_OH and C_30_H_61_OH monolayers are about 7–8 °C lower than those reported in previous studies.^84–86^ In our experiments, the monolayer surface area per droplet was about three times smaller than in previous studies, calculated by assuming spherical cap geometry and complete coverage of the air–water interface. This smaller surface area can account, at least in part, for the lower median freezing temperatures observed in our experiments.

**Fig. 5:**
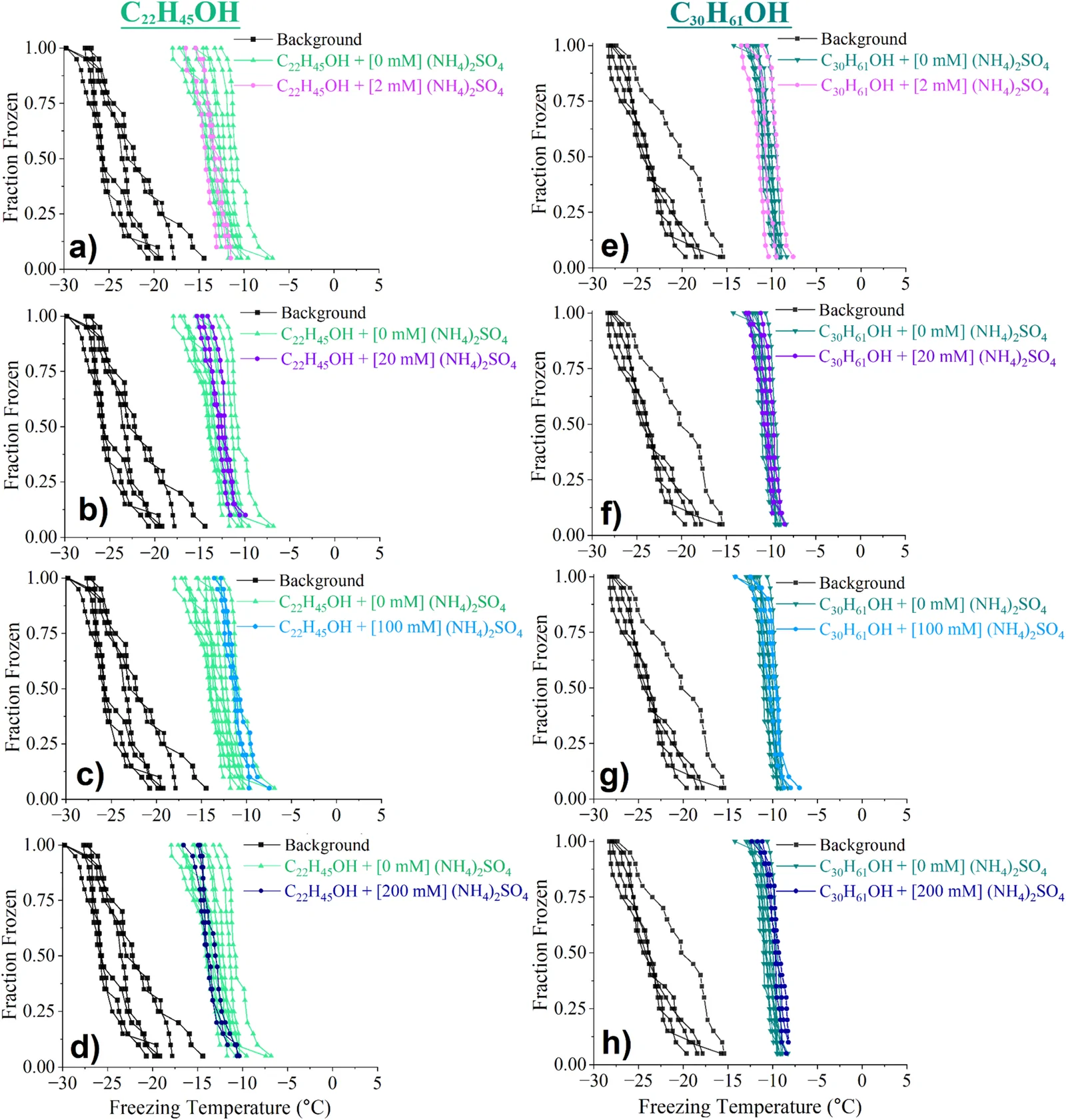
Freezing curves for aqueous ammonium sulfate droplets coated with C_22_H_45_OH (left panel) and C_30_H_61_OH (right panel): (a and e) 2 mM ammonium sulfate, (b and f) 20 mM ammonium sulfate, (c and g) 100 mM ammonium sulfate, and (d and h) 200 mM ammonium sulfate. For comparison, the curves for Milli-Q water droplets with C_22_H_45_OH and C_30_H_61_OH at 0 mM are shown in green and teal, respectively. The freezing curves for the background (water with 100 mM ammonium sulfate) are shown in gray. Freezing temperatures are corrected for freezing point depression.

The freezing curves for aqueous ammonium sulfate droplets coated with C_22_H_45_OH and C_30_H_61_OH monolayers closely resemble those of pure water droplets coated with the same monolayers after freezing point corrections (Fig. 5). Across all ammonium sulfate concentrations, the Δ*T*_50_ values were indistinguishable from zero within the measurement uncertainty (Fig. 3c and d), indicating that the effect of ammonium sulfate on the freezing properties of the monolayers was smaller than the experimental uncertainty.

Fatty alcohol monolayers also expose hydroxy groups at the interface, resulting in polar surfaces. Molecular dynamics simulations have shown that their ice nucleation ability is strongly governed by lattice matching with ice, with improved lattice alignment leading to higher nucleation efficiency.^72,73^ Since these monolayers present hydroxylated, and thus polar, surfaces, we initially hypothesized that exposure to ammonium cations would enhance their ice nucleation ability, based on the mechanism proposed by Whale *et al.*^59^ to explain ammonium-enhanced ice nucleation by inorganic particles. However, our results demonstrate that this mechanism does not extend to fatty alcohol monolayers.

Our results for the aqueous ammonium sulfate droplets coated with alcohol monolayers are consistent with previous studies by Cantrell *et al.*, who found that the addition of ammonium sulfate did not affect the freezing temperatures of water droplets coated with C_16_H_33_OH, C_17_H_35_OH, C_25_H_51_OH or C_30_H_61_OH monolayers.^64^ In their study, 10 µL aqueous ammonium sulfate droplets were coated with one of the monolayers, and the water activity of the aqueous droplets was varied from 0.99–0.86, which corresponds to ammonium cation concentrations of 0.26–3.2 M, based on an aerosol thermodynamic model.^67,96^

### 3.3 Microplastics (PET fibers and LDPE spheres)

**Fig. 6:**
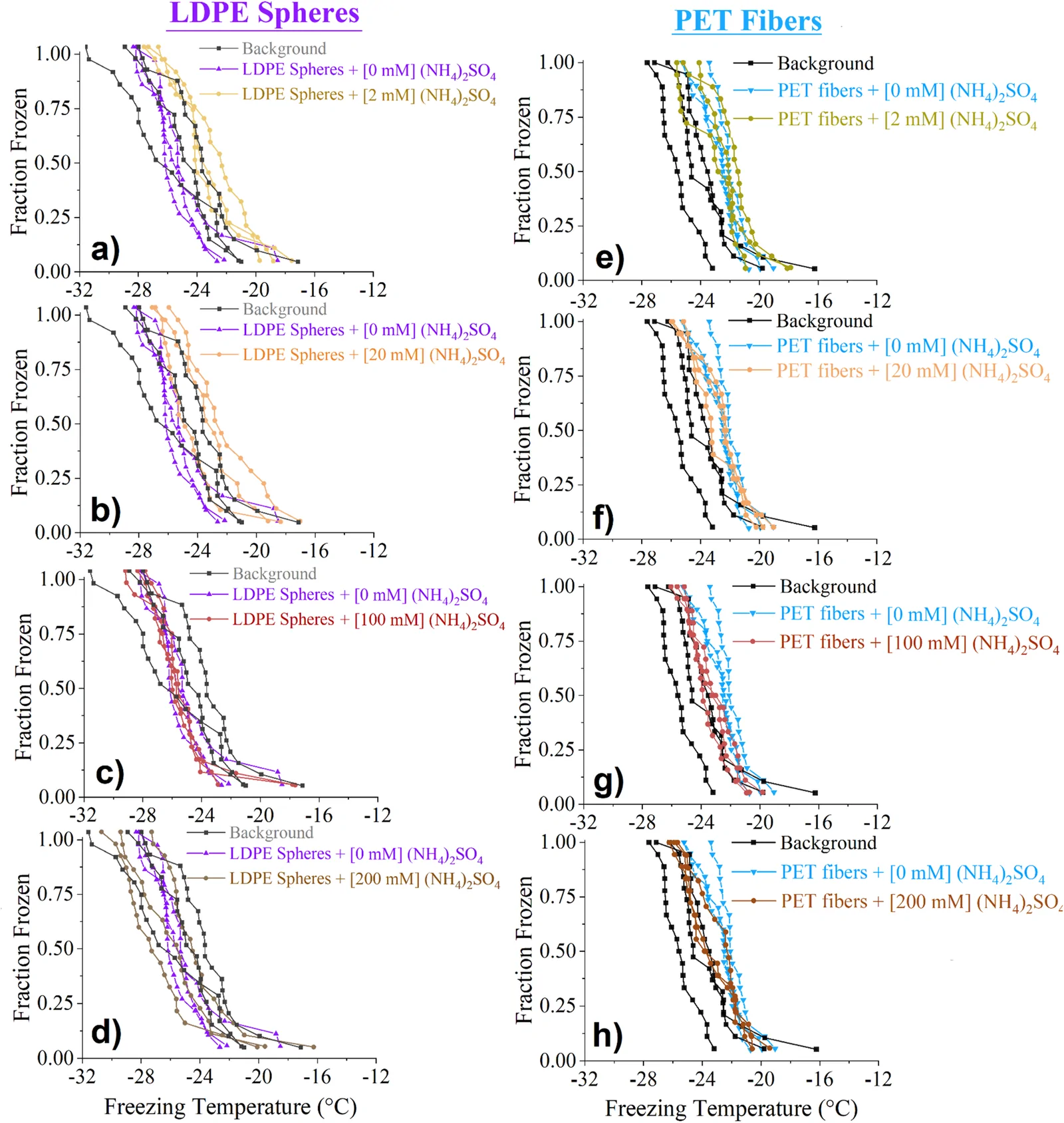
Freezing curves for aqueous ammonium sulfate droplets containing LDPE spheres (left panel) and PET fibers (right panel): (a and e) 2 mM ammonium sulfate, (b and f) 20 mM ammonium sulfate, (c and g) 100 mM ammonium sulfate, and (d and h) 200 mM ammonium sulfate. For comparison, the curves for Milli-Q water droplets with LDPE and PET at 0 mM are shown in purple and blue, respectively. The freezing curves for the background (water with 100 mM ammonium sulfate) are shown in gray. Freezing temperatures are corrected for freezing point depression.

We also performed freezing measurements of pure water droplets and aqueous ammonium sulfate droplets containing two types of microplastics: PET fibers and LDPE spheres. For pure water droplets with PET fibers and LDPE spheres, the measured *T*_50_ values were −22.3 °C ± 0.8 (*p* = 0.01) and −25.8 °C ± 0.7 (*p* = 0.01), respectively (Fig. 3e and f). These values agree with previously reported *T*_50_ values for pure water droplets containing the same types and concentrations of microplastics.^26^ It is important to note that the freezing curves for the pure water droplets with LDPE spheres overlapped with the background freezing curves (Fig. 6). In other words, when the experimental uncertainty is considered, the background and sample freezing spectra are not statistically distinguishable. Consequently, for aqueous ammonium sulfate droplets containing LDPE spheres, we can only determine whether the addition of ammonium sulfate increases freezing above the background level.

The freezing curves for aqueous ammonium sulfate droplets with PET fibers and LDPE spheres overlapped with those of pure water droplets containing the same microplastics (Fig. 6). Across all ammonium sulfate concentrations, the Δ*T*_50_ values were not statistically different from zero at the 99% confidence level (Fig. 3e and f). Based on this observation, we conclude that the freezing behavior of PET fibers and LDPE spheres was not significantly affected by the presence of ammonium sulfate within the uncertainty of the measurements. The uncertainty, reflected in the 99% confidence intervals, arose from day-to-day and experiment-to-experiment variability. Although the average Δ*T*_50_ values for LDPE spheres were positive at all concentrations, the corresponding 99% confidence intervals included zero in all cases, indicating that these positive values do not provide statistically significant evidence for an ammonium sulfate effect.

For PET, the results indicate that any effect of ammonium sulfate on freezing behavior are smaller than the measurement uncertainty. For LDPE, the results indicate that the additional of ammonium sulfate does not increase freezing above background by more than the measurement uncertainty.

In a previous study, Busse *et al.* investigated the effect of ammonium sulfate exposure on the freezing properties of several types of microplastics, including PET and LDPE particles. In their experiments, the microplastics were exposed to a 5 mM ammonium sulfate solution for 72 hours, then rinsed and filtered with UHPLC water to remove any residual ammonium sulfate before performing freezing droplet experiments in pure water.^27^ In contrast to our findings, they observed a decrease in the ice nucleation temperature for PET (Δ*T*_50_ = −2.8 °C ± 0.8, *p* = 0.05) and an increase for LDPE (Δ*T*_50_ = 4.2 °C ± 0.2, *p* = 0.05) following ammonium sulfate exposure.^27^

The differences between our results and those of Busse *et al.* may arise from the differences in exposure time used in the two experiments. In our study, the microplastics were in contact with the ammonium sulfate solution for only a few minutes prior to freezing, whereas Busse *et al.* exposed their samples for 72 hours. Additionally, the microplastics used in the two studies were obtained from different vendors and exhibited different morphologies, which may have contributed to the observed discrepancies.

A recent study by Bieber *et al.* showed that ice nucleation by microplastics occurs mainly at the plastic-water-air contact line.^97^ In other words, ice nucleation occurs at the plastic–water–air interface rather than within the bulk water or on the fully immersed plastic surface. Possible explanations for this result are as follows. Curvature at the contact line can create negative Laplace pressures,^98^ which can cause water to freeze at higher temperatures.^99^ Negative pressure can also influence the surface free energy between ice and water, which is required for ice nucleation.^100^ In addition, line tension at the contact line could enhance ice nucleation.^101^ Based on our measurements and this previous study, short-term exposure to ammonium sulfate may not affect the negative Laplace pressure or the line tensions at water–microplastic surfaces. This is consistent with ammonium sulfate having only a small effect on the surface tension of water and on the water contact angle at the low concentrations^1,102^ used in our experiments.

## 4 Conclusions and implications

### 4.1 Predicting ice nucleation in the atmosphere

Accurate predictions of ice cloud formation in the atmosphere require understanding how ammonium sulfate affects the freezing behavior of atmospheric INSs. Our results show that ammonium sulfate coatings increase the freezing temperature of submicrometer α-alumina particles. Submicrometer α-alumina has been proposed as a candidate material for stratospheric aerosol injection to offset warming driven by CO_2_ emissions.^68,69^ In the stratosphere, injected α-alumina particles would become coated with sulfuric acid and could later be transported to the troposphere, where exposure to ammonia would partially or fully neutralize the coating, forming ammonium sulfate.^68–70^

Once in the troposphere, these particles can be incorporated into cloud droplets in mixed-phase clouds. Consistent with previous studies, we show that submicrometer α-alumina can nucleate ice in mixed-phase clouds, which can modify cloud properties and influence wet deposition, affecting the atmospheric residence time of α-alumina. Since ammonium sulfate shifts ice nucleation by α-alumina to warmer temperatures, ammonium sulfate coatings on α-alumina could promote ice formation in mixed-phase clouds at warmer temperatures, potentially increasing cloud glaciation and precipitation. Enhanced precipitation would also increase the likelihood of α-alumina removal by wet deposition. These processes should be considered when assessing the atmospheric impacts and fate of α-alumina proposed for stratospheric aerosol injection and solar radiation management.

In contrast, the organic materials studied here show no measurable change in freezing temperature after short-term exposure, on the order of minutes, to ammonium sulfate. This suggests that, on short timescales, ammonium sulfate coatings are unlikely to alter how the microplastics studied here (PET and LDPE) influence mixed-phase cloud properties, precipitation, or long-range transport. However, our experiments address only short exposure times. Longer exposure on the order of 72 hours may affect the ice nucleation properties of these microplastics.^27^ Additional work is needed to bridge the gap between our short-term experiments and long-term exposure studies.

As mentioned above, the freezing curves for pure water droplets containing LDPE spheres overlapped with the background freezing curves (Fig. 6a–d). Consequently, for LDPE spheres, we could only determine whether the addition of ammonium sulfate increased freezing above the background level. Therefore, it remains possible that ammonium sulfate influenced the freezing temperature of LDPE spheres, but that the change was outside our detection range. Specifically, the change may have occurred at temperatures below the freezing temperatures of pure water droplets (*T*_50_ values of approximately −26 °C) in our experiments. Experiments that can probe lower freezing temperature ranges (*T*_50_ values < −26 °C) would therefore be useful for better quantifying the effect of ammonium sulfate on LDPE.

Interestingly, the freezing properties of supermicrometer α-alumina were unaffected by short-term exposure to ammonium sulfate. This size-dependent response is consistent with a difference in surface hydroxy protonation state. Surfaces with more deprotonated, negatively charged hydroxy groups can attract ammonium cations, altering interfacial hydrogen-bonding networks and stabilizing nascent ice clusters, thereby enhancing ice nucleation.

### 4.2 Freezing assay for detecting mineral dust in atmospheric samples

A freezing assay was recently proposed and applied by our group to identify ice-nucleating mineral dust in atmospheric samples based on their short-term response to ammonium sulfate exposure.^55,103,104^ In this method, an increase in freezing temperature after adding ammonium sulfate indicates the presence of certain ice-nucleating minerals, such as K-feldspar, kaolinite and montmorillonite. However, the accuracy of this approach relies on the assumption that short-term exposure to ammonium sulfate does not increase the freezing temperatures of organic materials in atmospheric samples.

Worthy *et al.*^55^ examined the effects of ammonium sulfate (with exposure times of a few minutes, similar to those in the present study) on various organic ice-nucleating substances, including humic acid, bacteria, fungi, and sea surface microlayer material, and found no enhancement in freezing temperature for any of them. Our results for alcohol monolayers and microplastics further support this finding, showing that short-term exposure to ammonium sulfate does not alter the freezing properties of organic materials found in the atmosphere. In other words, our results provide further support for the use of ammonium sulfate-based freezing assays for identifying certain types of ice-nucleating minerals, such as K-feldspar, kaolinite, and montmorillonite, within atmospheric samples. However, it should be kept in mind that not all mineral dust INPs are expected to respond to the ammonium sulfate assay. In addition, our results for α-alumina demonstrate that minerals with the same bulk composition can exhibit different responses to ammonium sulfate. These differences are most likely due to variations in surface properties between the samples. Since surface properties can depend on particle size (*e.g.*, for commercial α-alumina samples), the ammonium sulfate assay may, in some cases, be sensitive only to certain particle sizes. Consequently, the assay is expected to provide only a lower limit to the abundance of mineral dust INPs in atmospheric samples. Nevertheless, this information remains valuable because the abundance and concentration of mineral dust INPs in the atmosphere are still highly uncertain.

## Conflicts of interest

There are no conflicts to declare.

## Supplementary Material

EA-006-D5EA00157A-s001

## Data Availability

Data for this article including freezing droplet and fraction frozen data are available at the open science framework repository: short-term exposure to ammonium sulfate modifies ice nucleation by alpha-alumina but not organic monolayers or microplastics at https://doi.org/10.17605/OSF.IO/GR6NS Supplementary information (SI) is available. See DOI: https://doi.org/10.1039/d5ea00157a.
