# Supplementary material for: Short-term exposure to ammonium sulfate modifies ice nucleation by alpha-alumina but not organic monolayers or microplastics
Source: Environ Sci Atmos. 2026 Jul 8. Online ahead of print. doi: 10.1039/d5ea00157a (PMC13383377; doi:10.1039/d5ea00157a)
Supplement: EA-OLF-D5EA00157A-s001 [file EA-OLF-D5EA00157A-s001.pdf]

Supplementary Information (SI) for Environmental Science: Atmospheres.
This journal is © The Royal Society of Chemistry 2026

Supporting Information (SI):

Short−Term Exposure to Ammonium Sulfate Modifies Ice Nucleation by Alpha-Alumina but Not Organic Monolayers or Microplastics

Lian Pharoah, Teresa M. Seifried, Gren N. Patey, Anita Lam, and Allan K. Bertram*

Department of Chemistry, University of British Columbia, Vancouver, British Columbia, Canada V6T 1Z1

E-mail: bertram@chem.ubc.ca

The supporting information document has 10 pages consisting of 3 tables and 6 figures.

Table of Contents

Supplemental Note 1. SEM images

Table S1. SEM instrument details, experimental conditions, and sample preparation

Figure S1. SEM images of the supermicrometer (a, b, c) and submicrometer (d, e, f) $\alpha-Al_2O_3$ powders

Supplemental Note 2. XRD data analysis

Table S2. XRD instrument details, experimental conditions, and sample preparation

Figure S2. XRD spectrum of the submicrometer $\alpha-Al_2O_3$ powder

Figure S3. XRD spectrum of the supermicrometer $\alpha-Al_2O_3$ powder

Figure S4. XRD Rietveld data fitting of the submicrometer $\alpha-Al_2O_3$ powder with the calculated profile of aluminum borate structure[1] $9Al_2O_3 \cdot 2B_3O_4$ (A_9B_2) using PDF-2 in HighscorePlus

Figure S5. XRD Rietveld data fitting of the submicrometer $\alpha-Al_2O_3$ powder with the calculated profile of aluminum borate structure[2] $Al_{18}B_4O_{33}$ using PDF-2 in HighscorePlus

Figure S6. XRD Rietveld data fitting of the supermicrometer $\alpha-Al_2O_3$ powder using PDF-2 in HighscorePlus

Supplemental Note 3. BET analysis

Table S3. BET instrument details, experimental conditions, and sample preparation

S1. SEM.

SEM was carried out to determine the size and morphology of the α−Al_2O_3 powders. Table S2 includes details on the instrument, experimental conditions and sample preparation. Figure S6 shows SEM images of the samples.

Table S1: SEM preparation

Instrument	Zeiss XB350 Field Emission
Resolution	Ultrahigh up to 0.7 nm
Electron Gun	Field Emission Schottky gun - Low voltage
Sample	Dry α−alumina powders deposited on individual aluminum SEM stubs (25 mm diameters) coated with adhesive carbon tape. Each sample was dispersed onto a glass slide and then were picked up with the stub using the carbon tape as an adhesive
Coater and Dryer	Samples were then sputter coated with Platinum using the Leica EM ACE600 coater
Acceleration Voltage Used	4.00 kV
Detector	Secondary Electron Detector

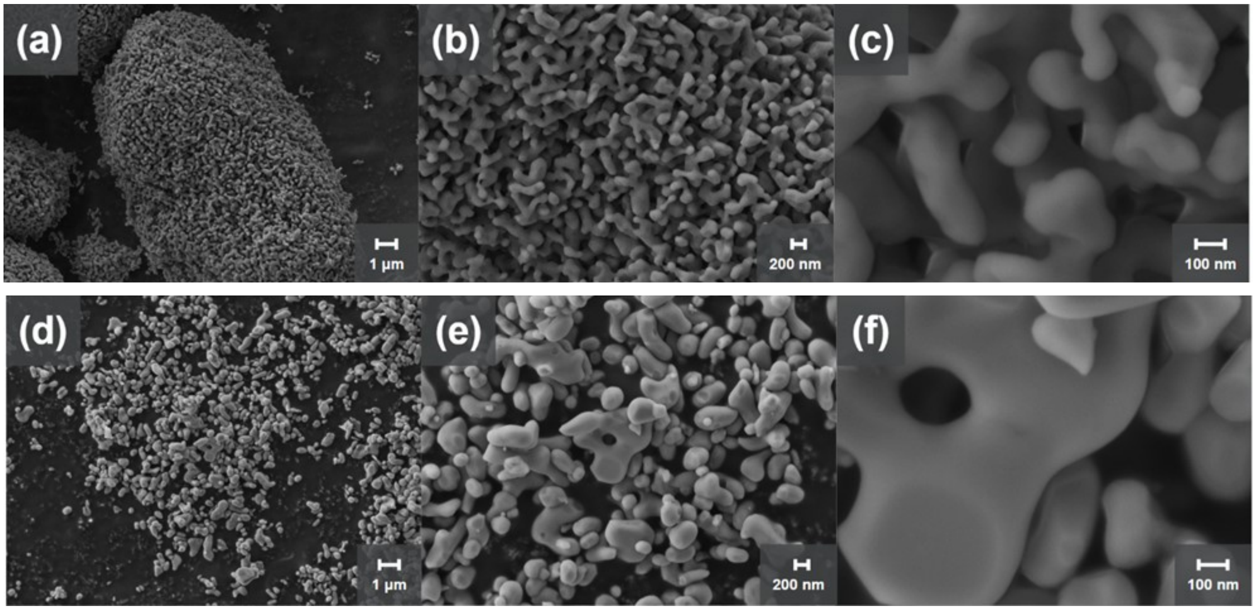

Figure S1: Scanning Electron Microscopy images of the supermicrometer $\alpha-Al_2O_3$ powder at different magnifications (a) 5.0x, (b) 15.0x, (c) 75.0x, and of the submicrometer $\alpha-Al_2O_3$ powder at (d) 5.0x (e) 15.0x, (f) 75.0x magnification.

S2. XRD measurements.

XRD measurements were carried out on the α-alumina ($\alpha-Al_2O_3$) samples to determine the chemical composition of the samples. Table S1 lists the experimental details of the measurements, and Figures S1−S2 show XRD analysis of the submicrometer and supermicrometer $\alpha-Al_2O_3$ samples, respectively. Figures S3−S4 show XRD Rietveld analysis, which fits experimental XRD data to calculated profiles using PDF-2 in HighscorePlus, of the submicrometer $\alpha-Al_2O_3$ sample. In addition, XRD results with calculated profiles of aluminum borate structures (A_9B_2 and $Al_{18}B_4O_{33}$) are added to the graphs, respectively. The aluminum borate structure, $Al_{18}B_4O_{33}$[2] modelled in Figure S4 fits the composition of the submicrometer $\alpha-Al_2O_3$ sample better than the one using the Garsche structure (A_9B_2)[1]. Both aluminum borate structure models suggest that the submicrometer $\alpha-Al_2O_3$ sample is approximately 95% $\alpha-Al_2O_3$ and 5% aluminum borate.[1,2] Figure S5 shows the XRD Rietveld analysis of the supermicrometer $\alpha-Al_2O_3$ sample, which shows 100% $\alpha-Al_2O_3$.

Table S2: XRD Instrument Details

Instrument	Bruker D8-Advance X-Ray Diffractometer
Configuration	Bragg-Brentano
Radiation	Copper Kα_1 & Kα_2
Filter	Nickel (filters out CuKβ)
Detector	LynxEye Silicon Strip
Generator	40 kV, 40 mA
Slits	0.6 mm divergent, 8 mm anti-scatter, 2.5°, soller
Sample	Powder, packed in standard Bruker sample holder; not rotated
Software	Bruker software was used. HighscorePlus was used with the ICDD PDF-2 database for phase ID. TOPAS was used was used for Rietveld modeling. CIF files were obtained from the American Mineralogist structure database and the Crystallography Open Database (COD).

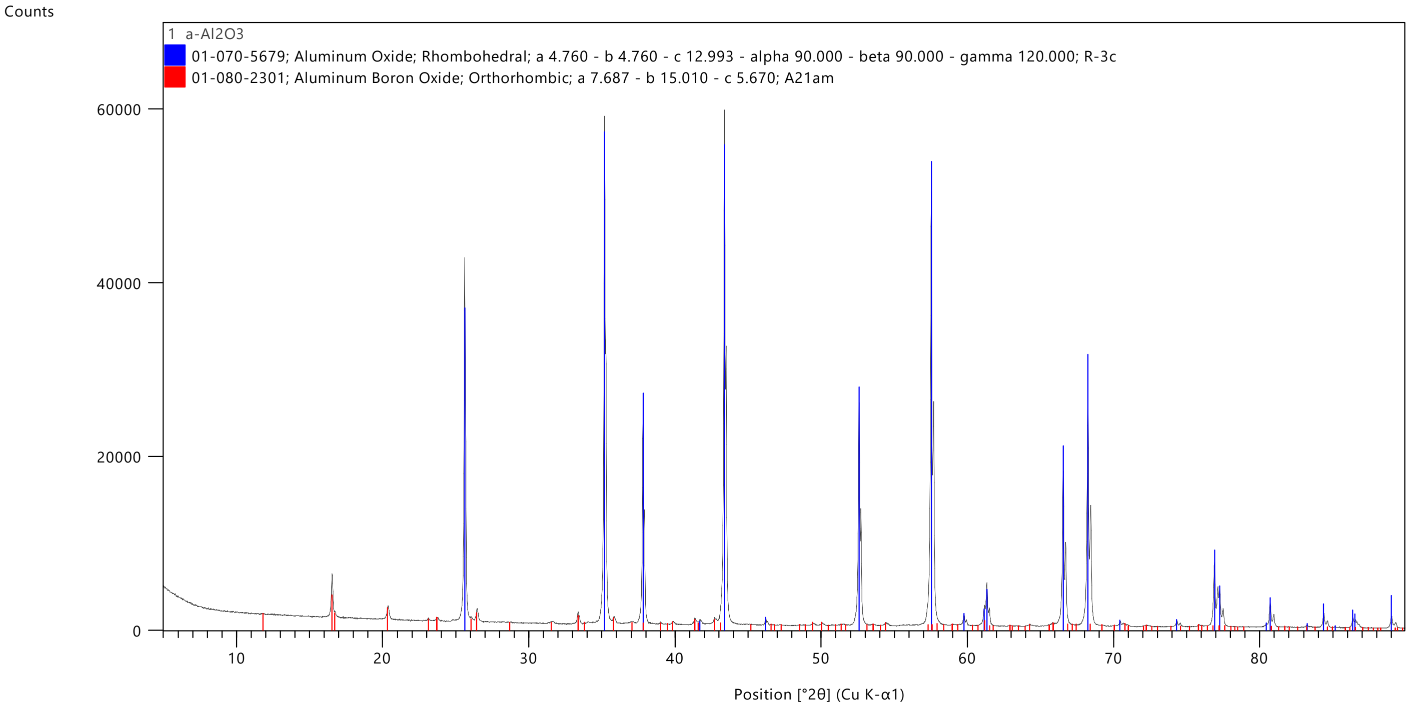

Figure S2: XRD analysis of the submicrometer $\alpha-Al_2O_3$ powder (black line). For comparison, corundum synthetic Al_2O_3 and aluminum boron oxide XRD profiles are included colored in blue and red, respectively

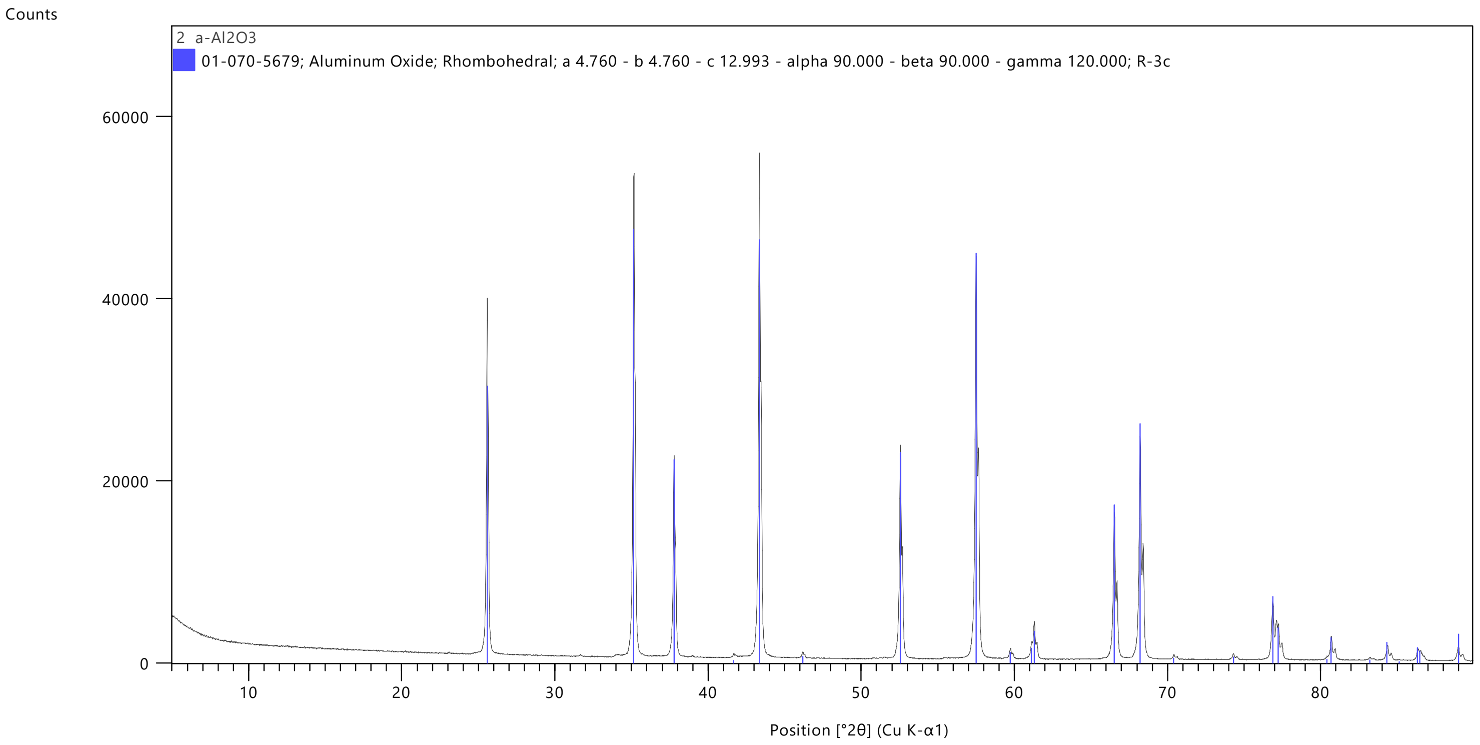

Figure S3: XRD analysis of the supermicrometer $\alpha-Al_2O_3$ powder (black line). For comparison, corundum synthetic Al_2O_3 is included colored in blue.

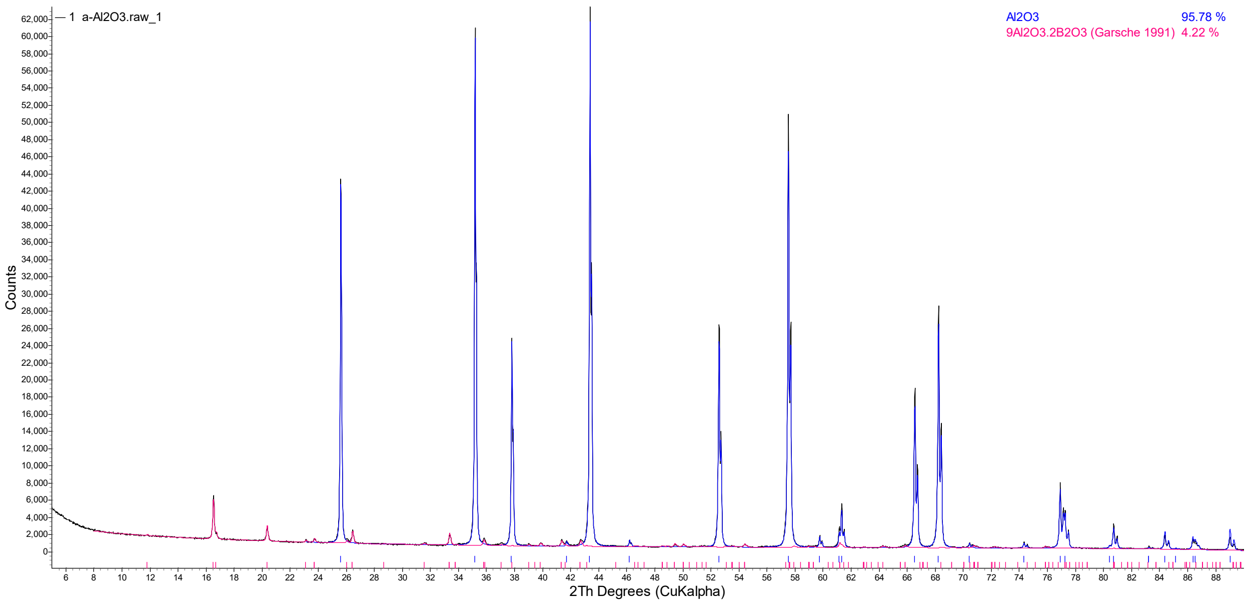

Figure S4: XRD Rietveld analysis of the submicrometer α−Al_2O_3 powder. Al_2O_3 is depicted in blue and the aluminum borate structure $9Al_2O_3{\cdot}2B_3O_4$ (A_9B_2) in pink.[1] The aluminum borate structure used here is the same as the one matched using PDF-2 in HighscorePlus.

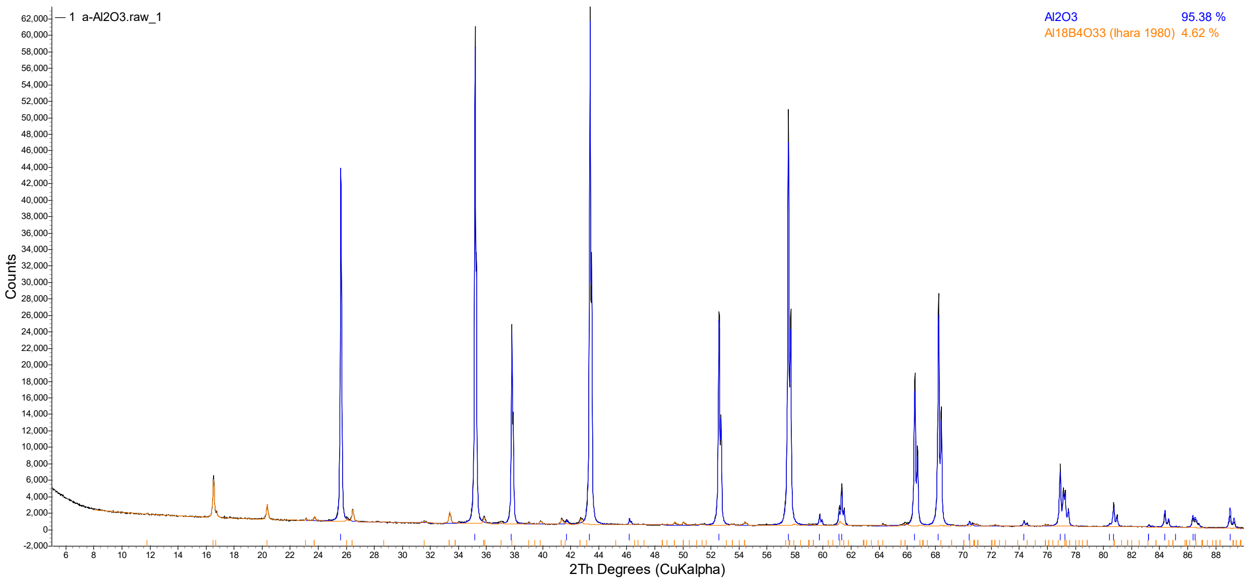

Figure S5: XRD Rietveld analysis of the submicrometer α− Al_2O_3 powder. Al_2O_3 is depicted in blue and the aluminum borate structure $Al_{18}B_4O_{33}$ in orange.[2] The aluminum borate structure used here has a similar unit cell to the one reported by Garsche et al. 1991[1] that was matched from PDF-2 in HighscorePlus. This model gave a slightly better fit than the one using the Garsche structure.[1]

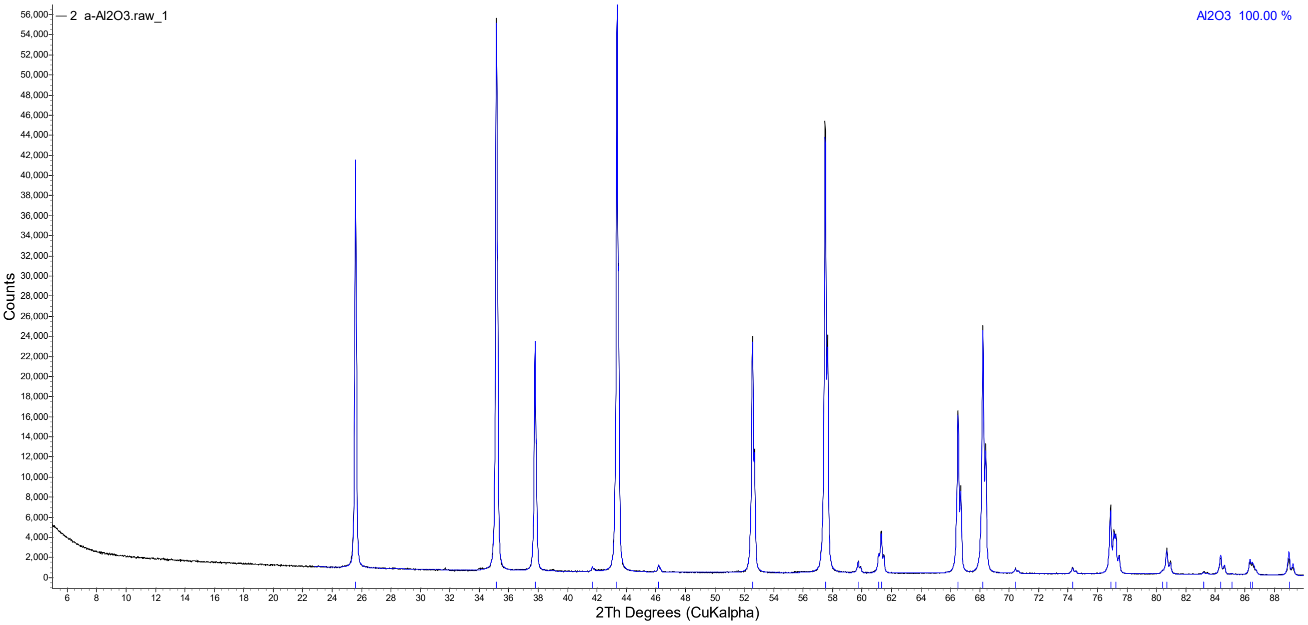

Figure S6: XRD Rietveld analysis of the supermicrometer $\alpha-Al_2O_3$ powder.

S3. BET.

BET[3] was carried out to determine the surface area of the two $\alpha-Al_2O_3$ powders. Table S3 includes details on the instrument used for the measurements, experimental conditions and sample preparation.

Table S3: BET Analysis Details[4]

Instrument	Nitrogen sorption isotherms were collected at 196 K on a Micrometrics accelerated surface area and porosimetry (ASAP) 2020 analyzer
Samples	1.272 g – Submicrometer $\alpha-Al_2O_3$<br>1.171 g – Supermicrometer $\alpha-Al_2O_3$
Degassing	Degassed at 120 °C for 12 hrs under the following conditions:<br>Evacuation phase: 10 °C/min, 120 °C, 6 hrs<br>Heating phase: 10 °C/min, 120 °C, 6 hrs

Detector	Brunauer–Emmett–Teller (BET) method for surface area analysis<br>Barrett–Joyner–Halenda (BJH) method for pore size analysis

Reference

(1) Garsche, M.; Tillmanns, E.; Almen, H.; Schneider, H.; Kupcik, V.; Incorporation of Chromium into Aluminum Borate $9Al_2O_3{\cdot}2B_3O_4$ (A_9B_2). Eur. J. Mineral. 1991, 3(5), 793−808.

(2) Ihara, M.; Imai, K.; Fukunaga, J.; Yoshida, N.; Crystal structure of Boroaluminate, $9Al_2O_3{\cdot}2B_2O_3$. J. Ceram. Soc. Japan, Yogyo-Kyokai-Shi. 1980, 88(2), 77−84.

(3) Brunauer, S.; Emmett, P. H.; Teller, E. Adsorption of Gases in Multimolecular Layers. *J. Am. Chem. Soc.* 1938, 60(2), 309– 319.

(4) Ren, Y. Exploration of Factors that Influence Heterogeneous Ice Nucleation: Laboratory Experiments and Molecular Dynamics Simulations. Ph.D. thesis, University of British Columbia, 2023.
